# Phagocytosis via Complement or Fc-Gamma Receptors Is Compromised in Monocytes from Type 2 Diabetes Patients with Chronic Hyperglycemia

**DOI:** 10.1371/journal.pone.0092977

**Published:** 2014-03-26

**Authors:** Blanca I. Restrepo, Marcel Twahirwa, Mohammad H. Rahbar, Larry S. Schlesinger

**Affiliations:** 1 Division of Epidemiology, UTHealth Houston, School of Public Health at Brownsville, Brownsville, Texas, United States of America; 2 Joslin Diabetes Center-Doctors Hospital at Renaissance, Edinburg, Texas, United States of America; 3 Division of Clinical and Translational Sciences, Department of Internal Medicine, Medical School, University of Texas Health Science Center at Houston, Houston, Texas, United States of America; 4 Center for Microbial Interface Biology and Department of Microbial Infection and Immunity, The Ohio State University, Columbus, Ohio, United States of America; University of Rochester, United States of America

## Abstract

Type 2 diabetes patients (DM2) have a higher risk of tuberculosis (TB) that may be attributed to functional defects in their mononuclear phagocytes given the critical role of these cells in *Mycobacterium tuberculosis* containment. Our previous findings suggest that monocytes from DM2 have reduced association with serum-opsonized *M. tuberculosis*. To determine if this alteration is due to defects in phagocytosis via complement or Fc-gamma receptors (FcγRs), in this study we evaluated the uptake of sheep red blood cells coated with IgG or complement, respectively, by monocytes from individuals with and without DM2. We found that chronic hyperglycemia was significantly associated with reduced phagocytosis via either receptor by univariable and multivariable analyses. This defect was independent of host serum opsonins and flow cytometry data indicated this was not attributed to reduced expression of these phagocytic receptors on DM2 monocytes. The positive correlation between both pathways (R = 0.64; p = 0.003) indicate that monocytes from individuals with chronic hyperglycemia have a defect in the two predominant phagocytic pathways of these cells. Given that phagocytosis is linked to activation of effector mechanisms for bacterial killing, it is likely that this defect is one factor contributing to the higher susceptibility of DM2 patients to pathogens like *M. tuberculosis*.

## Introduction

Diabetes mellitus is associated with compromised immunity and higher susceptibility to pulmonary infections, with the most common pathogens being *Mycobacterium tuberculosis*, *Staphylococcus aureus*, *Streptococcus pneumoniae* and *Klebsiella pneumoniae* [1]. Among these, tuberculosis (TB) is the number one bacterial killer worldwide with 1.4 million deaths annually [2], [3]. The increasing number of individuals with type 2 diabetes mellitus (DM2) in TB-endemic countries is a re-emerging concern for TB control [4]–[7]. The biological basis for the increased susceptibility to TB in DM2 patients is likely explained by their dysfunctional immunity [8]–[14]. Mononuclear phagocytes (monocytes and macrophages) play a major role in TB immunopathogenesis [15]. Monocytes phagocytose *M. tuberculosis* and allow for its intracellular replication or under appropriate stimuli, contain its growth [16], [17]. Monocytes also migrate to tissue sites during *M. tuberculosis* infection and here they contribute to the local response. Given the importance of monocytes in *M. tuberculosis* biology it is likely that functional alterations in these cells in DM2 patients will contribute to their increased susceptibility to TB, or their poorer prognosis once TB develops [18].

We recently found that monocytes from DM2 patients who are *M. tuberculosis*-naïve have reduced association (binding and/or phagocytosis) of *M. tuberculosis* when compared to non-DM2 monocytes. This difference between study groups was observed in-vitro with higher autologous serum concentrations (p = 0.03 with 20% serum vs p = 0.25 with 5% serum) [8], suggesting a defect in *M. tuberculosis* entry that is mediated by serum opsonins (e.g. C3b/iC3b and/or natural IgG) and/or their corresponding complement (CRs) and FcγRs on DM2 monocytes. Further experiments using *M. tuberculosis* opsonized with heat-inactivated serum suggested the defect in DM2 is attributed to a heat-labile component. This led us to suspect that DM2 patients have alterations in complement that impair *M. tuberculosis* binding and phagocytosis by monocytes: the defect could be in DM2 monocyte receptors (CR1, CR3) and/or their serum ligands (C3b, iC3b).

The goal of the present study was to determine whether monocytes from DM2 patients have defects in the expression or function of the major receptors for opsonin-dependent phagocytosis. The complement receptor-mediated entry of ligands was our prime suspect, but given that CR3 may operate with other receptors to mediate phagocytosis, we also evaluated the function of the other major class of phagocytic receptors that synergize with complement receptors on human monocytes, the FcγRs (CD64, CD32 and CD16) [19]. We assessed whether functional defects found in monocytes from DM2 patients were attributed to lower surface expression of CD11b (the α chain of CR3), and/or FcγRs, or a reduced proportion of blood monocyte subsets that are highly phagocytic [20].

## Materials and Methods

### Ethics Statement

This study was approved by the committee for the protection of human subjects of UTHealth (FWA00000667). Ethical guidelines of the Helsinki Declaration of 1964 and the US Department of Health and Human Services were followed, including signing of an informed consent by all study participants and treatment of data from interviews or laboratory findings as strictly confidential.

### Participant Enrollment and DM2 Classification

Healthy adults with or without DM2 were identified and enrolled as described previously [8]. Individuals were interviewed to assess their history of DM2 and other factors that could affect their immune response. Participants taking metformin or anti-inflammatory medications (e.g. corticosteroids, aspirin or TNF-α blockers) were excluded due to possible alterations in monocyte phenotype, and those with type 1 diabetes were excluded in order to focus the study on DM2. Height and weight were recorded to calculate body mass index (BMI) and those with a BMI higher than 40 were excluded. Blood was drawn to measure glucose (Accu-Chek, Roche), glycated hemoglobin (HbA_1c_; A_1c_ Now, Bayer), triglycerides and total and HDL cholesterol (PTS panels test strips, Cardiocheck). DM2 classification was based on the American Diabetes Association 2010 definition of self-reported diabetes or chronic hyperglycemia (HbA_1c_≥6.2%) [21]. Hyperglycemia was defined as a fasting glucose ≥126 mg/dl or random glucose ≥200 mg/dl. Controls without DM2 were selected on the basis of a normal HbA_1c_ (<6.2%), normoglycemia at enrollment and no self-reported diabetes.

### Monocyte Isolation, Culture and Storage

PBMCs were isolated from heparinized blood on a Ficoll-sodium cushion (GE Health, Piscataway, NJ). For flow cytometry, aliquots of PBMCs were stored frozen at −70°C in RPMI with 10% DMSO and 20% fetal bovine serum. For functional studies, the remaining fresh PBMCs were cultured in teflon wells (Savillex Corp., Minnesota, monocyte) with 20% autologous plasma (1.5–2.0×10^6^ cells/ml) [16]. After overnight incubation the PBMCs were washed, re-suspended in RPMI, 2% HEPES and 20% autologous plasma and transferred into 8-well glass chamber slides (Thermo Scientific, West Palm Beach, FL) at 2×10^6^ cells per well. After incubation for 2 h at 37°C in 5% CO_2_ the non-adherent cells were removed and the remaining adherent cells (≥90% monocytes by Giemsa staining) were evaluated for FcγR- and CR-mediated phagocytosis.

### Complement- or FcγR-mediated Phagocytosis Assays

Sheep red blood cells (sRBCs; Lampire Biologicals) were stained with the red fluorescent dye PKH26 (Sigma) and opsonized with C3b and iC3b or IgG. For coating with C3b/iC3b the stained sRBCs were incubated with anti-sheep RBC IgM (1∶2000) for 40 min at 37°C and then 10% C5-deficient serum (Sigma) for 20 min at 37°C. For IgG coating, the sRBCs were incubated with rabbit anti-sheep RBC stroma (Sigma; 1∶200) for 40 min at 37°C. For each study participant, non-opsonized sRBCs were included as a control. The opsonized (or non-opsonized) sRBCs in RPMI-HEPES were added to the adhered monocytes at a 10∶1 ratio and incubated for 30 min at 37°C. Phagocytosis was stopped by adding water for 45 sec to lyse non-phagocytosed RBCs and slides were mounted with an aqueous mounting medium containing DAPI for staining of monocyte nuclei. The number of monocytes with at least one internalized sRBC was calculated by counting >200 monocytes representing at least four 200x fields by fluorescence microscopy. Among monocytes with at least one sRBC, phagocytosis efficacy was evaluated as the percentage of monocytes with 1, 2, 3, 4 or more sRBCs. During assay development the specificity of CR-mediated phagocytosis was assessed by confirming the requirement for C3 fragment deposition from intact (versus heat-inactivated) serum (Fig. S1).

### Monocyte Characterization by Flow Cytometry

Frozen PBMCs were thawed and distributed into three tubes for antibody staining. All tubes contained antibodies to the following markers: CD14-FITC (Southern Biotechnology Associates), CD16-AF700 (BD Bioscience), and CD3- and CD19-PerCP.Cy5.5 (eBioscience). Additional antibodies included: Tube 1, CD11b-APC-Cy7 (eBioscience) and CD32-PE (BD Bioscience); Tube 2, CD64-PE (BD Bioscience); and Tube 3, CD32B-PE detected with goat anti-rabbit PE (AbCAM). Proper isotype controls were included for each primary antibody. All incubations were done in ice. Fc-receptors were blocked with human IgG (Sigma-Aldrich) for 20 min, followed by addition of antibodies for 30 min in PBS with 1% bovine serum albumin, 0.1 mM EDTA and 0.02% sodium azide. Prior to analysis 7AAD (BD Bioscience) was added to each tube. Acquisition was conducted in a FACS CANTO-II using FACS DIVA v6.0 (BD Biosciences). Monocytes were identified based on their forward and side scatter properties and then dead (7AAD-positive), CD3 and CD19-positive cells were excluded. Further details on the gating strategy are provided in the Supplement (Fig. S2). After identifying monocyte sub-populations based on CD14 and CD16 expression, the median fluorescence intensities of each fluorochrome was established.

### Statistics

Differences between study groups were evaluated by the chi square test (when the expected cell count for all cells were >5) or Fisher’s exact (when the expected cell count for least one of the cell count n ≤5) for dichotomous variables and t-test for continuous variables. Linear relationships were established by Pearsons correlations or linear regression models. For multivariable linear regression models we tested the underlying assumptions on the outcome variables including the normality and equal variance of the residuals. Variables with a p value ≤0.15 by univariable analysis were entered as a possible confounding factor for multivariable analysis using generalized linear models. P values ≤0.05 were considered significant, and values between 0.051 and 0.099 marginally significant.

## Results

### Host Factors Associated with Reduced Phagocytosis

Data from participants evaluated for FcγR-mediated phagocytosis (10 with DM2 and 16 without DM) are shown in Table 1. Most were Hispanic white females. Among the factors frequently associated with DM2 (older age, higher BMI and dyslipidemias), BMI was significantly higher and older age was marginally significant among DM2 patients. Among the participants with DM2, seven (70%) had poor glucose control. DM2 patients were more likely to have vision problems that cannot be corrected with lenses and self-reported frequent urination and excessive thirst. The monocytes from a subset of these participants (8 with DM2 and 11 without DM) were evaluated for CR-mediated phagocytosis and had similar characteristics to the 26 individuals evaluated for FcγR-mediated phagocytosis.

**Table 1:** Characteristics of participants for phagocytosis assays by type 2 diabetes status.

	All n = 26	Diabetes n = 10	No diabetes n = 16	P value
Sociodemographics:				
Age in years, mean(SD)	47 (11)	51 (7)	44 (13)	0.08
Female gender	17 (63%)	8 (80%)	9 (56%)	0.21
Hispanic White	21 (81%)	9 (90%)	12 (75%)	0.62
BCG vaccination	21 (81%)	8 (80%)	13 (81%)	1.00
Obesity and dyslipidemia:				
BMI, mean (SD)	29 (5)	32 (6)	27 (3)	**0.03**
Total cholesterol (mg/dL), mean(SD)	191 (46)	200 (60)	185 (36)	0.43
HDL cholesterol (mg/dL), mean(SD)	37 (9)	35 (6)	37 (10)	0.48
Triglycerides (mg/dL), mean(SD)	100 (61)	103 (68)	99 (59)	0.87
Diabetes control^1^				
Poor DM2	7 (27%)	7 (70%)	0 (0%)	**<0.001**
Good DM2	3 (11%)	3 (30%)	0 (0%)	
No DM2	16 (62%)	0 (0%)	16 (100%)	
Comorbidities:				
High blood pressure	4 (15%)	3 (30%)	1 (6%)	0.26
Vision problems	4 (15%)	4 (40%)	0 (0%)	**0.006**
Frequent urination	9 (35%)	7 (70%)	2 (13%)	**0.003**
Excessive thirst	7 (27%)	5 (50%)	2 (13%)	**0.04**

1 For diabetes control, poor = DM2 with HbA_1c_≥7% and hyperglycemia, and good = DM2 with HbA_1c_<7% and euglycemia; P values estimated by student *t* test for continuous variables, or chi-square for discrete variables (when cell count n>5) or Fisher’s Exact (when cell count n≤5); p values ≤0.099 have gray highlight and p values ≤0.05 are shown in bold; BMI, body-mass index; SD, standard deviation.

**Figure 1:**
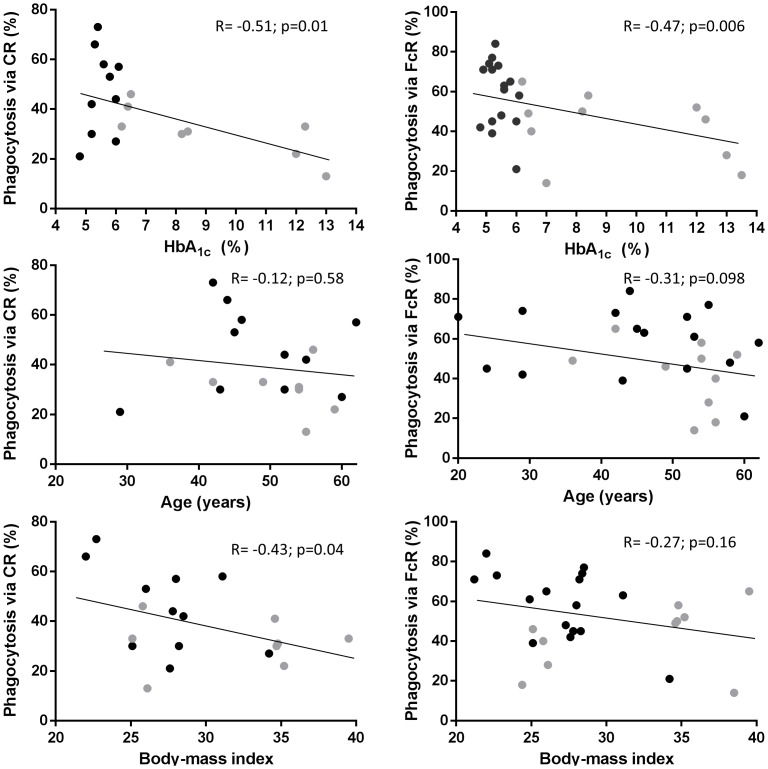
Correlations between FcγR- and CR-mediated phagocytosis and host characteristics. sRBCs were coated with C3b/iC3b or IgG and the percentage of monocytes with at least one phagocytosed sRBC was established by fluorescence microscopy among participants with and without DM2. The correlation between phagocytosis via CRs for C3b/iC3b-coated sRBCs and via FcγRs for IgG-coated sRBCs is shown with respect to HbA_1c_, or two other host characteristics that are also distinct in DM2 patients: age and BMI. Correlation coefficients (R) and corresponding p values are provided, and the linear regression displayed. Black circles, no DM2; gray circles, DM2.

To determine if DM2 is associated with reduced phagocytosis mediated by CRs or FcγRs, adherent monocytes from DM2 patients or controls were evaluated for the degree of overall phagocytosis of sRBCs coated with C3b/iC3b or IgG, respectively (% of monocytes with at least one phagocytosed RBC; Table 2; Fig. S3) and phagocytosis efficacy (number of sRBCs per monocyte; Fig. S4). Overall phagocytosis of non-opsonized sRBC controls ranged from 0.5–2% with no difference by DM2 status (data not shown). For CR-mediated phagocytosis, DM2 (versus no DM) had both lower overall phagocytosis (mean difference 14; p = 0.05; Table 2) and phagocytosis efficacy (Fig. S4). Accordingly, the degree of overall CR2-mediated phagocytosis was inversely associated with DM2-defining parameters like hyperglycemia and chronic hyperglycemia (β-coefficients = −0.14 and −3.17, respectively with both p<0.05; Table 2 and Fig. 1). Higher BMI was the only other host characteristic associated with reduced CR-mediated phagocytosis (β-coefficient = −1.42; p<0.05). Since participants with DM2 had higher BMI and tended to be older than those without DM (p≤0.15; Table 1), we controlled for these potential confounders and calculated adjusted mean differences or β-coefficients (Table 2). These adjusted estimates remained statistically significant for the inverse relationship between decreased CR-mediated phagocytosis and high blood glucose, HbA_1c_ levels or BMI (shown in bold in Table 2), but were no longer significant for DM2. Other host factors were not associated with altered CR-mediated phagocytosis after adjustment for the aforementioned covariates.

**Table 2:** Relationship between host characteristics and percent of MN phagocytosis of sheep red blood cells coated with antibodies or complement.

Host characteristics	% of monocytes with at least one ingested sRBC mediated by:							
	CRs				FcγRs			
Discrete variables:	n	mean (SD)	Mean difference(95% CL)	Adj mean difference(95% CL)	n	mean (SD)	Mean difference(95% CL)	Adj mean difference(95% CL)
Diabetes								
Yes	8	31 (10)	−14 (−28.9, 0.03)	−10.1 (−24.1, 3.8)	10	42 (17)	**−17 (−30.7, 2.4)**	−13.7 (−28.4, 1.1)
No	11	45 (17)			16	59 (17)		
Gender								
Female	12	37 (18)	−6.0 (−22.4, 10.3)	−4.4 (−17.70, 8.92)	17	50 (21)	−6.3 (−22.2, 9.6)	−9.3 (−23.5, 4.9)
Male	7	43 (13)			9	56 (12)		
**Continuous variables:**	**n**		**β-coef (95% CL)**	**Adj β-coef (95% CL)**	**n**		**β-coef (95% CL)**	**Adj β-coef (95% CL)**
Age, yrs	19		−0.23 (−1.07, 0.60)	−0.12 (−0.89, 0.64)	26		−0.50 (−1.10, 0.09)	−0.43 (−1.02, 0.17)
BMI	19		**−1.42 (−2.76, −0.08)**	**−1.39 (−2.74, −0.04)**	26		−0.99 (−2.37, 0.39)	−0.76 (−2.13, 0.61)
Blood glucose	19		**−0.14 (−0.24, −0.04)**	**−0.14 (−2.45, −0.05)**	26		**−0.12 (−0.24, −0.01)**	−0.11 (−0.22, 0.01)
HbA_1c_	19		**−3.17 (−5.59, −0.77)**	**−3.15 (−5.49, −0.81)**	26		**−3.32 (−5.67, −0.96)**	**−3.0 (−5.44, −0.46)**
Total cholesterol	19		0.01 (−0.15, 0.19)	0.05 (−0.11, 0.20)	26		−0.05 (−0.21, 0.10)	−0.04 (−0.19, 0.11)
HDL cholesterol	19		0.55 (−0.32, 1.42)	0.47 (−0.32, 1.26)	26		0.33 (−0.50, 1.18)	0.06 (−0.78, 0.90)
Triglycerides	19		0.03 (−0.10, 0.15)	0.09 (−0.30, 0.20)	26		−0.07 (−0.18, 0.04)	−0.03 (−0.15, 0.08)

Adj = values adjusted for age and BMI; SD, standard deviation; CRs = complement receptors CR1 and/or CR3; FcγRs = CD64 (FcγR-I), CD32 (FcγR-II) and CD16 (FcγR-III); p values ≤0.099 have gray highlight and p values ≤0.05 are shown in bold. Blood glucose, total cholesterol, high-density cholesterol and triglyceride values measured in mg/dL and HbA1c in % glycated hemoglobin.

**Figure 2:**
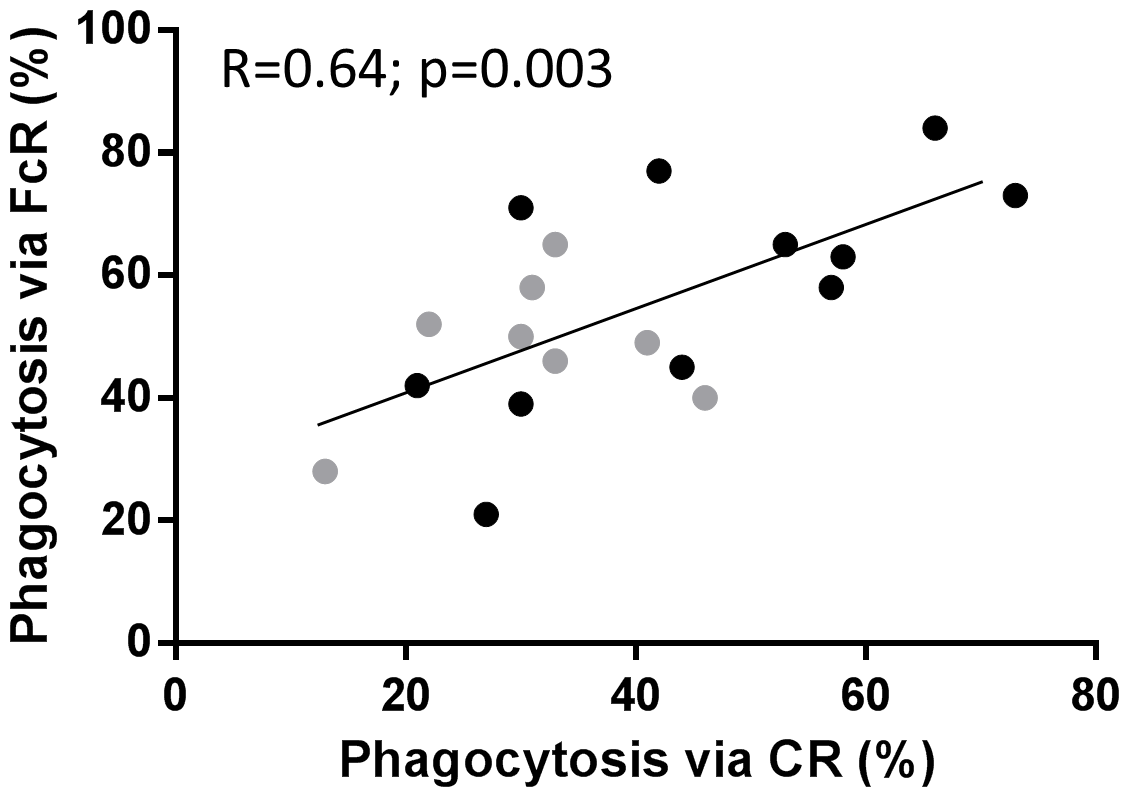
Correlation between CR- and FcγR-mediated phagocytosis. Phagocytosis of sRBCs coated with C3b/iC3b or IgG is described in the Methods and the linear relationship between both phagocytosis pathways is displayed. Correlation coefficients (R) and corresponding p value is provided. Black circles, no DM2; gray circles, DM2.

Phagocytosis mediated by FcγRs was lower in participants with DM2 (versus no DM) with a mean difference of 17 (p = 0.02; Table 2) for overall phagocytosis and fewer sRBCs per monocyte (Fig. S4). For the overall FcγR-dependent phagocytosis there was an inverse association with age (β coefficient = −0.50; p = 0.098) and the degree of hyperglycemia and chronic hyperglycemia (β coefficients = −0.12 and −3.32, respectively; p<0.05), but there was no association with other host factors (Table 2 and Fig. 1). After adjusting for age and BMI, FcγR-mediated phagocytosis remained lower in participants with DM2 (mean difference = 13.7; p = 0.07) and was inversely associated with chronic hyperglycemia (β coefficient = −3.0; p = 0.02) or hyperglycemia (β coefficient = −0.11; 0.08). Given the overall trend for reduced phagocytosis via FcγRs or CRs in DM2 patients with high HbA_1c_, we suspected that individuals with a defect in one mechanism would also have a similar defect in the other pathway. This was confirmed by the strong correlation between both mechanisms (R = 0.64; p = 0.003; Fig. 2).

### Phenotypic Characterization of Monocytes by Flow Cytometry

Human blood monocytes have variable phenotype and function and can be divided into subsets based on the expression of CD14 and CD16 [20], [22], [23]. The reduced phagocytosis of DM2 monocytes via CRs or FcγRs could be due to a lower proportion of the classical (CD14++CD16−) or intermediate (CD14++CD16+) subsets which are the most phagocytic when compared to the non-classical (CD14+CD16+) subset. Other possible explanations for lower phagocytosis in DM2 include reduced expression of CRs or FcγRs, up-regulated expression of CD32B which is an alternatively-spliced variant of CD32 that has an ITIM motif that reduces FcγR-mediated phagocytosis [24], and/or functional defects in phagocytosis. To explore the first three possibilities we conducted flow cytometry experiments to compare blood monocytes from DM2 versus no DM2 controls for differences in surface expression of the phagocytic receptors CD11b (the α chain of CR3), FcγR-I, -II and -III (CD64, CD32 and CD16, respectively), and CD32B. For these studies the sample size was expanded with monocytes from 17 additional participants enrolled in our previous study [8], for a total of 19 with DM2 and 24 controls. The sociodemographics of these participants are similar to those presented in Table 1.

**Figure 3:**
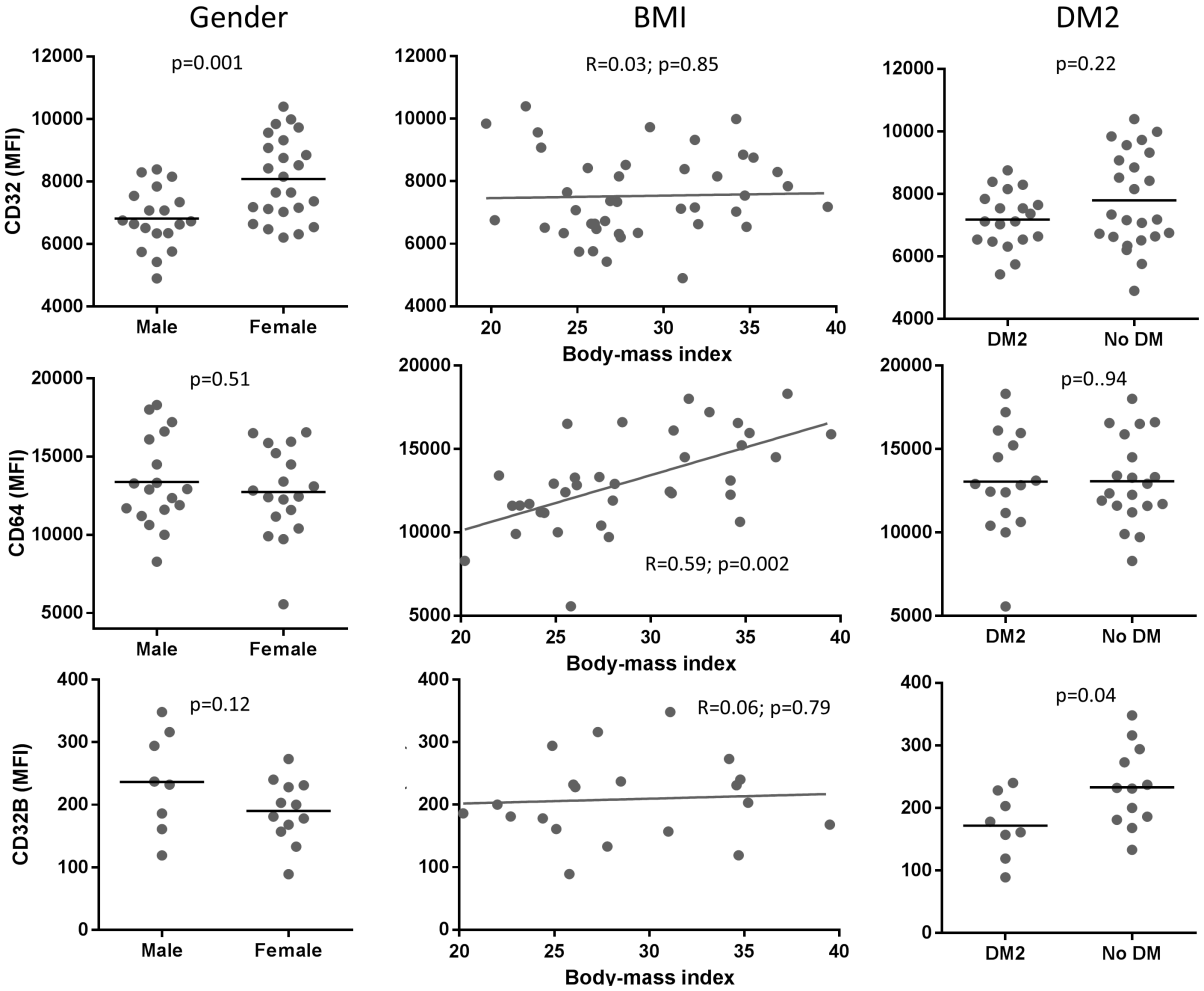
Relationship between host factors and the median fluorescence intensity (MFI) of phagocytosis-promoting and -inhibiting receptors. The mean of the MFI for monocyte receptors was established for all the CD14-positive monocytes or the classical, intermediate and non-classical monocytes as described in the Methods and shown in Table S2. Three host factors were associated with changes by univariable and multivariable analysis among all monocytes (gender, BMI and DM2 in columns), and their relationship with CD32, CD64 and CD32B is illustrated (rows): male gender with decreased CD32 but not CD64 or CD32B, higher BMI with increased CD64 but not CD32 or CD32B, and DM2 with increased CD32B expression. Correlation coefficients (R) and corresponding p values are provided, and the linear relationship displayed for BMI. Horizontal line shows mean by DM2 status.

First we assessed if there were variations in the proportion of classical, non-classical and intermediate monocyte subsets by DM2 status or other host characteristics. We found no differences by DM2, HbA_1c_ or glucose levels by univariable or multivariable analysis (Table S1). Among the other host characteristics only older age was associated with a shift from a lower proportion of classical to a higher proportion of non-classical monocytes. Second, we determined if the expression of phagocytic receptors (FcγRs and CD11b) was associated with DM2, high HbA_1c_, glucose levels or other host characteristics. Data are shown for all of the CD14-positive monocytes or stratified by classical, intermediate or non-classical subsets (Table S2). The only association between DM2 characteristics and phagocytic receptors was noted for CD32, which tended to be lower in individuals with DM2 (by multivariable analysis only) or in those with hyperglycemia by univariable analysis only. HbA_1c_ was not associated with any changes. Gender and BMI were associated with changes in some FcγRs, with similar findings in all of the CD14-positive monocytes and the classical subset. Male participants had lower CD32 expression and BMI was positively associated with CD64 by univariable and multivariable analysis (Fig. 3). Age only had a borderline correlation with reduced CD64 expression by univariable analysis. None of the evaluated host factors were associated with CD11b expression. Together, these findings suggested that gender and BMI influence CD32 and CD64 expression, respectively, while DM2 has a minor impact on FcγRs. Finally, we evaluated whether DM2 or other host characteristics were associated with increased expression of the phagocytosis inhibitory receptor CD32B. Unexpectedly, DM2 was the only host factor significantly associated with reduced (rather than increased) expression of CD32B by univariable and multivariable analyses (Table S2; Fig. 3). In summary, the monocytes from DM2 patients did not display changes in cell surface expression that would explain their reduced phagocytic capacity via FcγRs or CR3. Rather our data support the possibility that there is a functional defect in CR- and FcγR-mediated phagocytosis.

## Discussion

The few studies with monocytes from diabetes patients in human and murine models have consistently shown reduced phagocytosis of particles (latex beads) and various micro-organisms: *Burholderia pseudomallei*, *Listeria monocytogenes*, *Candida albicans*, *Escherichia coli and M. tuberculosis* [8], [25]–[27]. However, few studies have been conducted with human monocytes or with only DM2 patients (versus type 1 diabetes). Furthermore, it has been unclear whether reduced phagocytosis is attributed to factors in the monocyte itself and/or defects in serum opsonins. Our previous study in DM2 patients with *M. tuberculosis* suggested that reduced phagocytosis is more evident under higher serum conditions, which led us to search for defects in the major phagocytic receptors for opsonized pathogens: FcγRs and CRs [8], [28]. In the present study we show that human DM2 monocytes have a functional defect in phagocytosis mediated by CRs and FcγRs that is correlated with chronic hyperglycemia. This defect is independent of host serum or plasma opsonins, and not attributed to a reduced expression of CR3, FcγR-I, -II or -III, or to increased expression of the inhibitory phagocytic receptor CD32B.

Our functional studies with ligand-coated sRBCs provide support for the previous observation of reduced entry of *M. tuberculosis* into DM2 monocytes. Given that opsonin-dependent entry into monocytes is linked to activation of effector mechanisms for bacterial killing such as expression of pro-inflammatory cytokines, reactive oxygen species and lysosomal enzymes [29], it is likely that this defect contributes to the higher susceptibility of DM2 patients to pathogens like *M. tuberculosis*. However, the impact of our results on the intracellular fate of *M. tuberculosis* will need further investigation given that this bacterium normally replicates following phagocytosis. To date, the relationship between *M. tuberculosis* receptor usage and its survival within phagocytes is a lesser understood aspect of TB pathogenesis, especially for phagocytes from diabetes patients. For *M. tuberculosis*, entry of macrophages via the mannose receptor (which are not expressed in monocytes) is associated with reduced phagosome-lysosome fusion and increased PPARγ, the latter is a negative regulator of pro-inflammatory responses [30], [31]. For other intracellular pathogens like *Leishmania major, Salmonella typhi, Listeria monocytogenes* or *Francisella tularensis*, there is additional support for alternative outcomes of infection based on receptor usage. For example, entry of *L. major* or *S. typhi* via CR1 (but not CR3) avoids the activation of NADPH oxidase or of phagolysosome fusion, respectively [32]. For *F. tularensis*, entry via the C3 opsonin, CR3 pathway is associated with inhibition of TLR signaling [33]. It is possible that DM2 monocytes have functional defects in the receptors that enable *M. tuberculosis* entry, thereby promoting its intracellular survival as suggested in a study with *B. pseudomallei*. Like *M. tuberculosis*, the phagocytosis of *B. pseudomallei* is also lower in phagocytes isolated from diabetes models (peritoneal or bone marrow-derived macrophages from diabetic mice) [34]. However, once ingested, this bacterium has higher intracellular survival within the diabetic versus control macrophages. We are exploring whether this outcome is similar in human monocytes for *M. tuberculosis*.

The phenotypic characterization of monocytes by flow cytometry did not explain the lower phagocytosis of DM2 versus control monocytes. We explored several possibilities: First, there could be a lower proportion of phagocytic monocytes (classical) in DM2, but this was not observed. Second, DM2 monocytes could have lower expression of CR3 as suggested previously [35], or of FcγRs. However, there was only a trend towards lower CD32 in DM2. Third, DM2 monocytes could express higher levels of CD32B, an alternatively-spliced variant of CD32 that has an ITIM motif that reduces FcγR-mediated phagocytosis [24], but the opposite was observed: lower CD32B in DM2. This lack of relationship between monocyte phenotype and reduced phagocytosis in DM2 may be attributed to several factors: First, there may be defects in the expression of other receptors that mediate phagocytosis and were not evaluated in this study (*e.g.* CR1, TLRs, or CD36). Second, there may be functional defects in the receptors evaluated or in some downstream factors involved in phagocytosis. The process of phagocytosis involves many proteins and high energy expenditure [29], [36]. The strong inverse correlation we observed between phagocytosis via FcγRs and CRs, and high HbA_1c_ suggests that chronic hyperglycemia may lead to generalized defects in phagocytosis. This is consistent with previous studies where defects in monocyte phagocytosis have been reported for various opsonized microorganisms as well as one study with unopsonized latex beads in diabetic mice [27]. Our observation of reduced CD32B expression in DM2 provides further support for a generalized defect in the phagocytosis process. Furthermore, the absence of this “brake” on the immune response provides additional support for the increased pro-inflammatory phenotype in DM2 patients that is consistent with our previous work [9].

Our findings suggesting defects in the monocyte itself do not rule out the additional contribution of altered serum opsonins to reduced phagocytosis of opsonized *M. tuberculosis* or other microorganisms. In our previous work, there was a more notable defect in *M. tuberculosis* association with monocytes in 20% but not 5% autologous serum from DM2 patients. Other studies support defects in serum. Hyperglycemia has been suggested to cause structural changes in C3b that lead to reduced phagocytosis of *E. coli* [37] in DM2 patients and antibodies show significantly higher glycation when compared to non-DM2 controls but the impact of these findings on function is unclear [38], [39]. Among Hispanics, those with DM2 had lower antibody titers and reduced opsonophagocytosis of *S. pneumoniae* [40].

We recognize the potential study limitations, including the relatively small sample size of the functional phagocytosis assays which were sufficient to detect differences between DM2 and controls, but not to identify further interaction effects with other host variables. Studies in DM2 patients are inherently compromised by the presence of other co-morbidities and medications to manage them. We excluded individuals taking metformin given its impact on immunity [38], [39], but not those taking insulin, statins or other medications to manage their DM2 or other diseases which could have a variable impact on phagocytosis [41], [42].

In summary, our findings indicate that reduced phagocytosis via CRs and FcγRs is attributed at least in part to defects inherent to the DM2 monocyte itself. Future studies are warranted to determine the receptor-associated intracellular pathways involved and the additional contribution of serum opsonins from DM2 patients. Furthermore, it will be important to assess the relationship between the reduced phagocytosis by DM2 monocytes and the coupled activation of effector mechanisms for bacterial killing in these patients.

## Supporting Information

Figure S1 **Assessment of complement deposition and CR-dependent phagocytosis of sRBCs.**
(DOCX)

Figure S2 **Monocyte gating strategy.**
(DOCX)

Figure S3 **Representative fluorescent microscopy of phagocytosis of sRBCs coated with Ig, C3 or none by monocytes from DM2 and controls.**
(DOCX)

Figure S4 **Phagocytosis efficacy.**
(DOCX)

Table S1 **Relationship between the monocyte subset frequency and host characteristics.**
(DOCX)

Table S2 **Relationship between monocyte surface markers and the characteristics of individuals with and without DM2.**
(DOCX)
