# Supplementary material for: Phagocytosis via Complement or Fc-Gamma Receptors Is Compromised in Monocytes from Type 2 Diabetes Patients with Chronic Hyperglycemia
Source: PLoS One. 2014 Mar 26;9(3):e92977. doi: 10.1371/journal.pone.0092977 (PMC3966862; doi:10.1371/journal.pone.0092977)
Supplement: Figure S1 — Assessment of complement deposition and CR-dependent phagocytosis of sRBCs. (DOCX) [file pone.0092977.s001.docx]

**Figure S1. Assessment of complement deposition and CR-dependent phagocytosis of sRBCs**. sRBCs were incubated with anti-sheep RBC IgM (1:2000) for 40 min at 37°C and after washing the sRBCs were incubated for 20 min at 37°C with 10% C5-deficient serum (Sigma) that was fresh or heat-inactivated (HI, 56°C for 30 min). A. Aliquots of the sRBCs treated for both conditions (fresh or HI C5-negative sera) were lysed, proteins were reduced and denatured (boiling in the presence of SDS and 2-mercaptoethanol), separated by SDS-PAGE and evaluated for C3 deposition by Western blot with anti-C3 (MP Cappel). B. Aliquots of sRBCs incubated with fresh or heat-inactivated sera were added to adherent monocytes from a healthy control at a 10:1 ratio and incubated for 30 min at 37°C. Phagocytosis (% of monocytes with at least one RBC) was evaluated by fluorescence microscopy as described in the Methods.

**
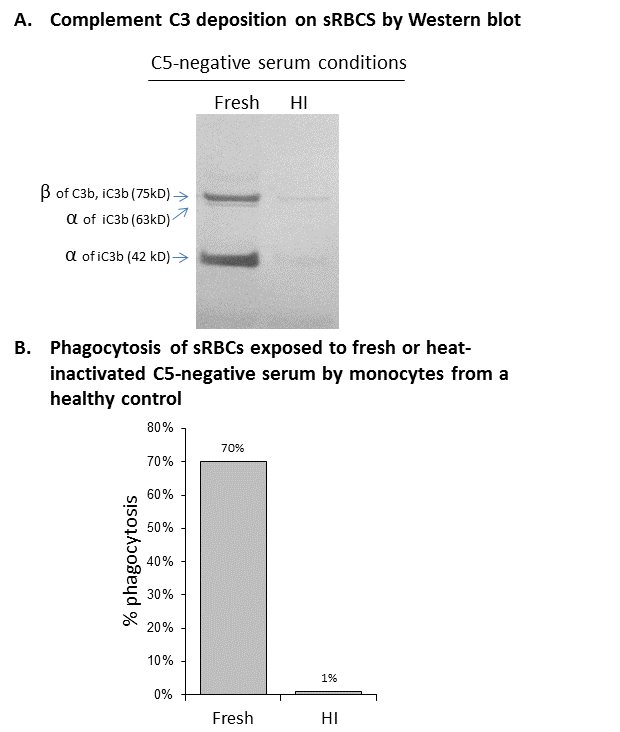
**
