# Supplementary material for: Phagocytosis via Complement or Fc-Gamma Receptors Is Compromised in Monocytes from Type 2 Diabetes Patients with Chronic Hyperglycemia
Source: PLoS One. 2014 Mar 26;9(3):e92977. doi: 10.1371/journal.pone.0092977 (PMC3966862; doi:10.1371/journal.pone.0092977)
Supplement: Figure S2 — Monocyte gating strategy. (DOCX) [file pone.0092977.s002.docx]

**Figure S2. Monocyte gating strategy**. During acquisition of PBMCs with FACS DIVA a threshold was set to exclude dead cells, platelets and debris and the monocyte subset was identified based on their scatter properties (A). Then the following cells were excluded: dead cells (7AAD-positive) and CD19- and CD3-positive lymphocytes stained with PerCP.Cy5.5 (B). At this point we ensured participants had more than 10,000 live monocytes for inclusion in the study. Further gating was conducted to exclude doublets or cell aggregates based on FSC-A by FSC-H properties (C) and CD14-/CD16- cells (D). The FCS files with these monocyte populations were then exported to FlowJo, where a cut-off value for CD14 and CD16 was established based on histograms of all the study participants to define the final cut-off for CD14+/CD16+, CD14++/CD16+ and CD14++/CD16- subpopulations for analysis (E). The remaining CD14-/CD16- cells were excluded from final analysis (F). The median fluorescence intensity of each of the fluorochromes for each marker was established for all the monocytes, or each of their three subpopulations.

**
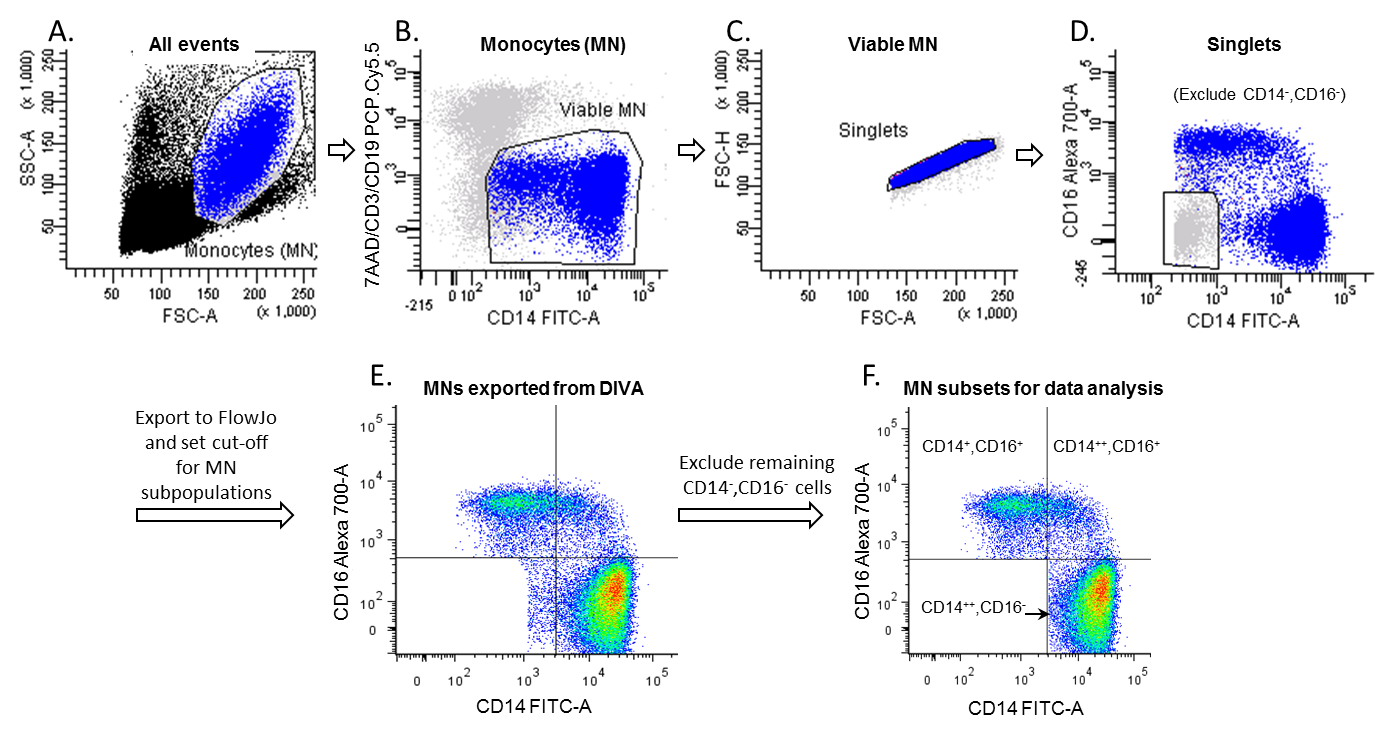
**
