# Supplementary material for: Phagocytosis via Complement or Fc-Gamma Receptors Is Compromised in Monocytes from Type 2 Diabetes Patients with Chronic Hyperglycemia
Source: PLoS One. 2014 Mar 26;9(3):e92977. doi: 10.1371/journal.pone.0092977 (PMC3966862; doi:10.1371/journal.pone.0092977)
Supplement: Figure S3 — Representative fluorescent microscopy of phagocytosis of sRBCs coated with Ig, C3 or none by monocytes from DM2 and controls. (DOCX) [file pone.0092977.s003.docx]

**Fig S3 Representative fluorescent microscopy of phagocytosis of sRBCs coated with Ig, C3 or none by monocytes from DM2 and controls.** sRBCs were fluorescently-labeled with PKH26 (red), coated with Ig, C3 or PBS (negative control) and incubated with adherent monocytes from individuals with and without DM2. Non-phagocytosed sRBCs were then lysed and the percentage of monocytes (nuclei stained with DAPI; blue) containing at least one phagocytosed sRBC was established by fluorescence microscopy (200x magnification). Details provided in the text.


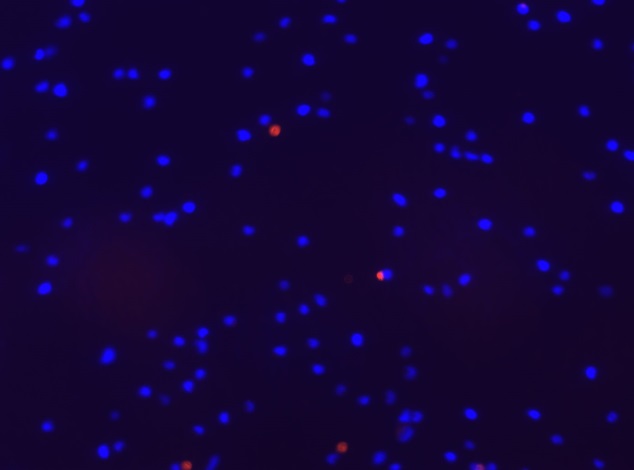

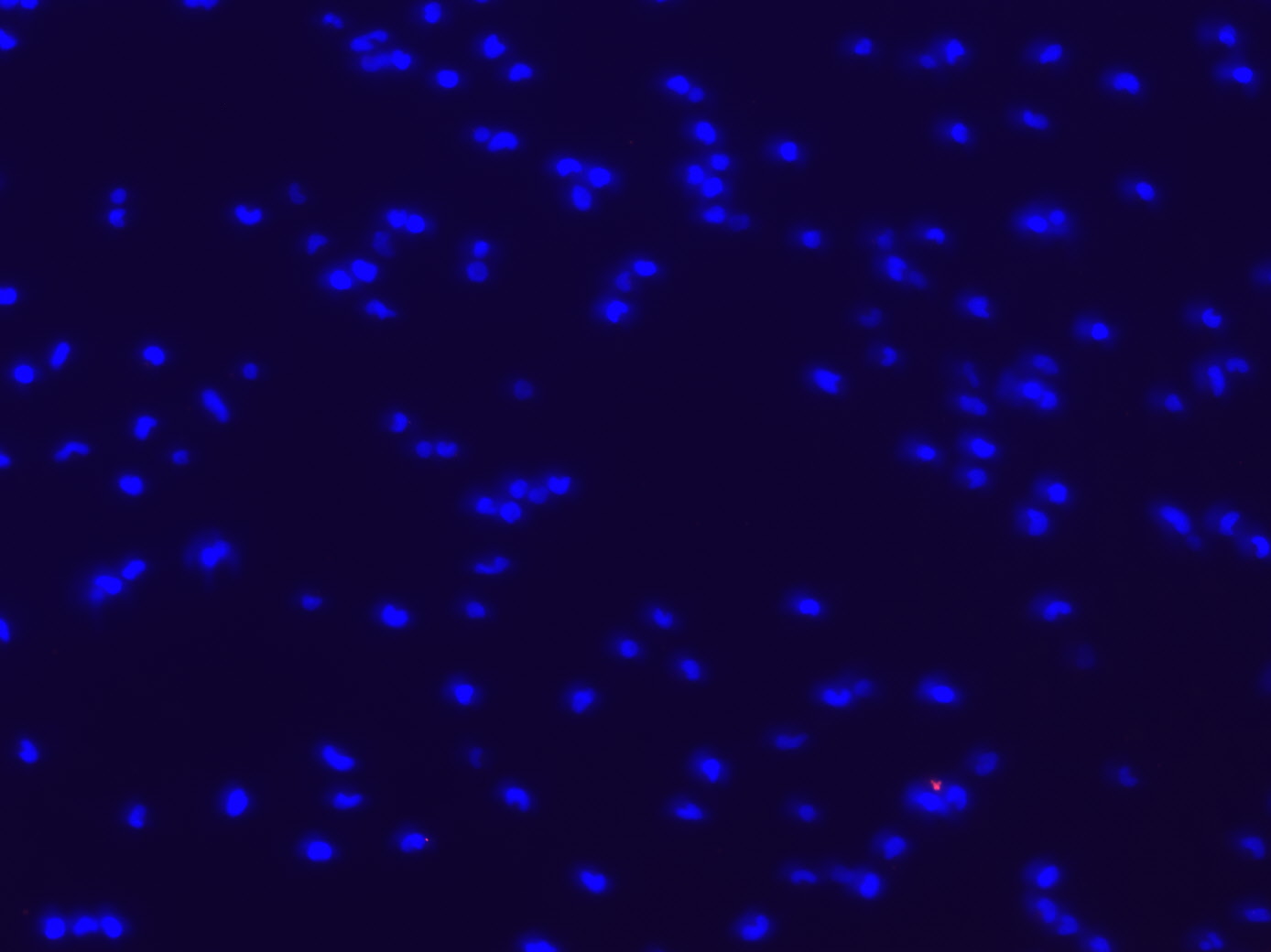

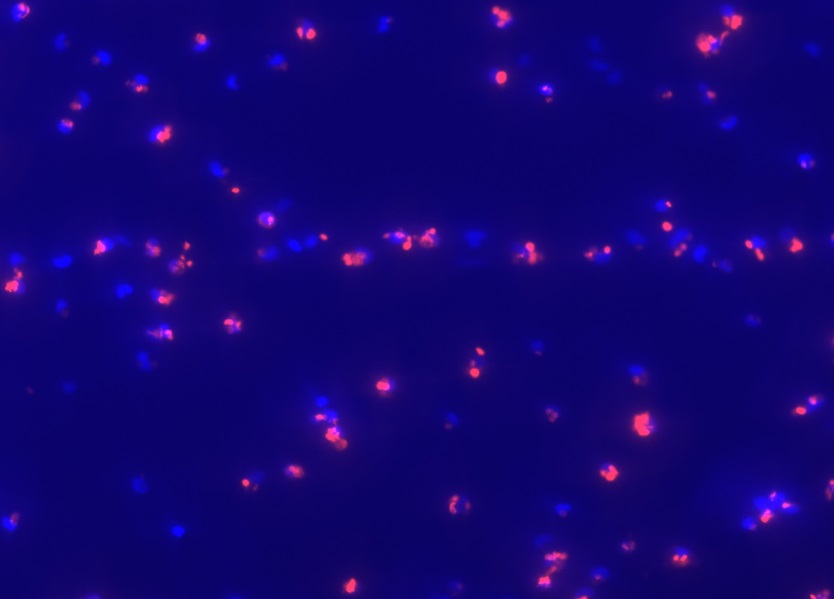

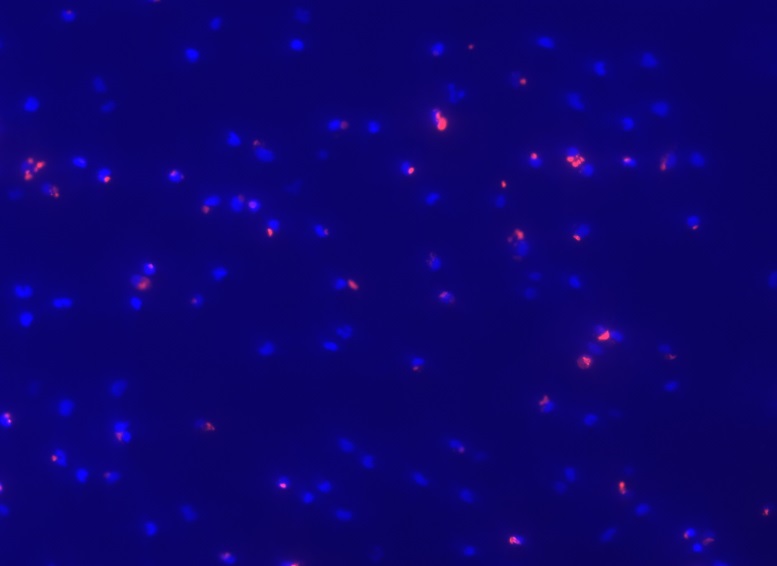

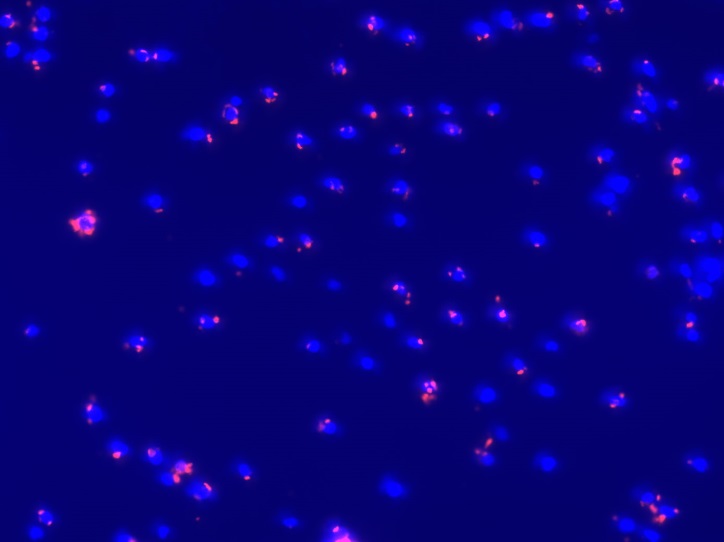

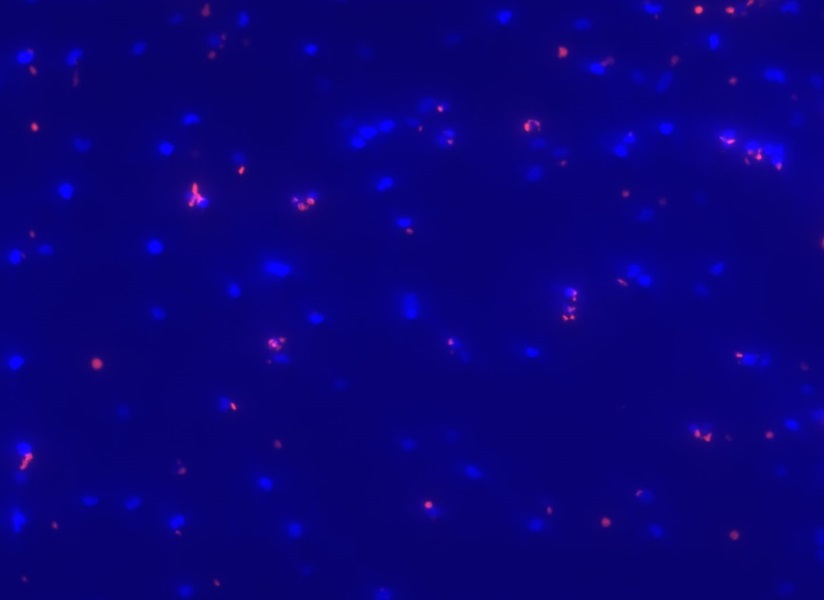


A. Ig-coated sRBCs, No DM2 monocyte

B. Ig-coated sRBCs, DM2 monocytes

C. C3-coated sRBCs, No DM2 monocyte

D. C3-coated sRBCs, DM2 monocytes

F. Non-coated sRBCs, DM2 monocytes

E. Non-coated sRBCs, no DM2 monocytes
