# Supplementary material for: Phagocytosis via Complement or Fc-Gamma Receptors Is Compromised in Monocytes from Type 2 Diabetes Patients with Chronic Hyperglycemia
Source: PLoS One. 2014 Mar 26;9(3):e92977. doi: 10.1371/journal.pone.0092977 (PMC3966862; doi:10.1371/journal.pone.0092977)
Supplement: Figure S4 — Phagocytosis efficacy. (DOCX) [file pone.0092977.s004.docx]

**Fig S4. Phagocytosis efficacy.** Fluorescently-labeled sRBCs were coated with IgG or C3, exposed to adherent monocytes from individuals with and without DM2, and phagocytosis was assessed by fluorescence microscopy as described in the Methods. Among monocytes with at least 1 phagocytosed sRBC, we determined the % with 1, 2, 3, 4 or more sRBCs by DM2 status. For the IgG opsonin condition, the distinction between 3 and 4 or more sRBCs was unclear, so data for these two categories were merged. The means (bars), standard deviations (vertical lines) and p values are shown for sRBCs coated with serum complement (A) or IgG (B).

**
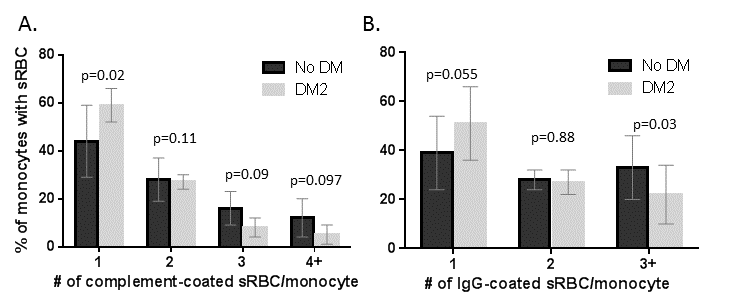
**
