# Supplementary material for: Phagocytosis via Complement or Fc-Gamma Receptors Is Compromised in Monocytes from Type 2 Diabetes Patients with Chronic Hyperglycemia
Source: PLoS One. 2014 Mar 26;9(3):e92977. doi: 10.1371/journal.pone.0092977 (PMC3966862; doi:10.1371/journal.pone.0092977)
Supplement: Table S1 — Relationship between the monocyte subset frequency and host characteristics. (DOCX) [file pone.0092977.s005.docx]

| **Table S1. Relationship between the monocyte subset frequency and host characteristics (n=43)** | | | | | | | |  |  |  |
| --- | --- | --- | --- | --- | --- | --- | --- | --- | --- | --- |
|  | Age | Gender | | |  | DM2 | | | HbA_1c_ | BMI |
| Monocyte subset |  | Male | Female | p |  | Yes | No | p |  |  |
|  | R (p) | Mean (SD) | Mean (SD) |  |  | Mean (SD) | Mean (SD) |  | R (p) | R (p) |
|  |  | n=19 | n=24 |  |  | n=19 | n=24 |  |  |  |
| % Classical | **-0.35 (0.03)** | 80.6 (5.8) | 81.7 (4.4) | 0.49 |  | 80 (5.2) | 82 (4.8) | 0.23 | -0.23 (0.17) | -0.07 (0.69) |
| % Intermediate | 0.01 (0.97) | 13 (4.4) | 11.8 (3.6) | 0.99 |  | 12.8 (3.4) | 12 (4.3) | 0.32 | 0.11 (0.52) | 0.19 (0.24) |
| % Non-classical | **0.35 (0.03)** | 5.5 (3.9) | 5.5 (2) | 0.35 |  | 6.1 (4) | 5.2 (2.1) | 0.53 | 0.14 (0.41) | -0.08 (0.61) |
| Significant differences shown in bold and gray highlight. BMI, body-mass index | | | | | | | | | | |
