# Supplementary material for: Phagocytosis via Complement or Fc-Gamma Receptors Is Compromised in Monocytes from Type 2 Diabetes Patients with Chronic Hyperglycemia
Source: PLoS One. 2014 Mar 26;9(3):e92977. doi: 10.1371/journal.pone.0092977 (PMC3966862; doi:10.1371/journal.pone.0092977)
Supplement: Supplementary file 6 [file pone.0092977.s006.docx]

Table S2. Relationship between monocyte surface markers and the characteristics of individuals with and without DM2 (n=43)[1]

	Sociodemographics					Diabetes							BMI		Adjusted P value[2]
	Age		Gender			DM2			HbA_{1c}		Blood glucose				
	R	P	Male MFI (SD)	Female MFI (SD)	P	Yes MFI (SD)	No MFI (SD)	P	R	P	R	P	R	P	
**CD14, all subsets**			n=19	n=24		n=19	n=24								
CD16	0.06	0.71	117 (30)	116 (25)	0.95	121 (34)	113 (22)	0.37	-0.07	0.67	0.08	0.61	0.16	0.32	
CD32	-0.07	0.65	6,804 (994)	8,102 (1,329)	**0.001**	7,208 (967)	7,740 (1,526)	0.22	-0.18	0.27	-0.25	0.11	0.03	0.85	Gender (0.001)
CD64	-0.29	0.08	13,378 (2,853)	12,745 (2,838)	0.51	13,046 (3,181)	13,075 (2,587	0.97	-0.03	0.84	0.02	0.93	0.59	**0.002**	BMI (<0.001)
CD11b	0.01	0.98	4,560 (629)	4,624 (1,333)	0.85	4,603 (1,096)	4,588 (1,040)	0.96	0.17	0.32	0.03	0.87	0.27	0.10	
CD32B	0.07	0.77	237 (79)	190 (50)	0.12	172 (52)	233 (64)	**0.04**	-0.27	0.25	-0.31	0.18	0.06	0.79	DM2 (0.052)
**C14++CD16- (Classical)**															
CD16	-0.11	0.50	80 (14)	91 (21)	0.07	86 (20)	86 (18)	0.94	-0.11	0.48	-0.01	0.96	0.09	0.56	Gender (0.09)
CD32	-0.13	0.41	6,408 (1,133)	7,646 (1,207)	**0.002**	6,848 (1,098)	7,264 (1,457)	0.32	-0.23	0.15	-0.28	0.07	0.13	0.40	Gender (<0.001); DM2 (0.06)
CD64	-0.21	0.20	13,839 (2,960)	14,047 (3,569)	0.85	13,986 (3,185)	13,904 (3,363	0.94	0.13	0.44	0.01	0.98	0.55	**<0.001**	BMI (<0.001)
CD11b	0.04	0.82	5,129 (788)	5,172 (1,299)	0.91	5,248 (1,134)	5,081 (1,047)	0.63	0.15	0.34	0.09	0.59	0.22	0.18	
CD32B	-0.06	0.80	194 (64)	154 (41)	0.09	142 (42)	192 (54)	**0.03**	-0.31	0.17	-0.38	0.09	0.08	0.74	DM2 (0.050)
**C14++CD16+ (Intermediate)**															
CD16	0.23	0.14	1,649 (345)	1,677 (415)	0.81	1,716 (336)	1,625 (417)	0.44	0.10	0.54	0.19	0.23	0.05	0.76	
CD32	0.18	0.25	13,490 (3,416)	14,746 (3,363)	0.23	14,302 (3,542)	14,075 (3,364	0.83	0.05	0.77	-0.08	0.63	0.36	**0.02**	BMI (0.02)
CD64	-0.19	0.24	5,156 (1,860)	5,950 (2,533)	0.28	5,860 (2,037)	5,276 (2,405)	0.43	0.14	0.42	0.09	0.61	0.38	**0.02**	BMI (0.04)
CD11b	0.12	0.45	3,460 (822)	3,655 (981)	0.50	3,666 (744)	3,486 (1,013)	0.54	0.05	0.76	0.01	0.95	0.23	0.16	
CD32B	-0.02	0.92	727 (225)	566 (202)	**0.09**	577 (183)	679 (246)	0.31	-0.22	0.35	-0.26	0.25	0.03	0.90	
**C14+CD16+ (Non-classical)**															
CD16	0.17	0.29	2,529 (550)	2,820 (551)	0.09	2,665 (489)	2,712 (625)	0.79	-0.01	0.93	0.01	0.97	0.13	0.41	
CD32	0.18	0.25	9,949 (3,189)	10,680 (3,654)	0.49	10,309 (3,376)	10,383 (3,550	0.95	0.11	0.49	-0.03	0.84	0.24	0.12	BMI (0.09)
CD64	0.02	0.90	1,220 (219)	1,377 (451)	0.18	1,361 (361)	1,242 (356)	0.32	0.25	0.14	0.2	0.23	0.14	0.41	
CD11b	0.02	0.91	526 (163)	521 (199)	0.93	529 (184)	519 (184)	0.86	0.08	0.62	0.01	0.99	-0.09	0.59	
CD32B	-0.07	0.75	770 (263)	623 (263)	0.22	602 (279)	749 (251)	0.22	-0.27	0.24	-0.32	0.16	-0.09	0.69	

[1] n=43 for all markers, except for CD11b (n=40), CD64 (n=39) and CD32B (n=23, 9 with DM2 and 12 without DM); Data is expressed as mean of the median fluorescence intensity (MFI), with MFI(SD) for discrete and the rho(p) value for continuous variables. p values ≤0.099 have gray highlight and p values ≤0.05 are shown in bold. [2]Multivariable model shows variables with $p < 0.1$ after controlling for age, gender, DM2 and BMI
